# Barriers and facilitators of abdominal aortic aneurysm screening in London: A cross-sectional survey

**DOI:** 10.1177/09691413241276187

**Published:** 2024-08-23

**Authors:** Ellie McKay, Joy Wong, Stella Ward, Josephine Ruwende, Robert Kerrison

**Affiliations:** 1 NHS England (London), London, UK; 2 Department of Behavioural Science and Health, 4919 University College London, London, UK; 3 School of Health Sciences, 3660 University of Surrey, Guildford, UK

**Keywords:** Abdominal aortic aneurysm, screening, barriers and facilitators, surveys

## Abstract

**Objectives:**

The aim of this research was to identify patient barriers and facilitators of abdominal aortic aneurysm (AAA) screening in London.

**Methods:**

A survey was distributed to 4211 adults, who had been invited for AAA screening in 2023. Barriers and facilitators were identified by comparing responses between attenders and non-attenders, using univariate logistic regression.

**Results:**

271 surveys were returned. Attendance was higher among respondents with a body mass index (BMI) > 25 (odds ratio [OR]: 2.72, 95% CIs [1.15, 6.46]; *p* < 0.05) and those with one or more comorbidities (OR: 3.82, 95% CIs [1.63, 8.98]; *p* < 0.01), but lower among those who had not visited a healthcare appointment within the past 6 months (OR: 0.41, 95% CIs [0.18, 0.94]). Attendance was also lower among those who believe screening is only useful for people with symptoms (OR: 0.37; 95% CIs [0.16, 0.89]; *p* < 0.05), find it difficult to make time for medical appointments (OR: 0.25, 95% CIs [0.10, 0.60]; *p* < 0.01), find it difficult to get to medical appointments (OR: 0.40, 95% CIs [0.17, 0.91]; *p* < 0.05), have more important medical problems to worry about (OR: 0.28, 95% CIs [0.12, 0.64]; *p* < 0.01), cannot afford to travel to medical appointments (OR: 0.16, 95% CIs [0.07, 0.38]; *p* < 0.001), need help getting to appointments (OR: 0.33, 95% CIs [0.13, 0.86]; *p* < 0.05), have caring responsibilities (OR: 0.15, 95% CIs [0.06, 0.34]; *p* < 0.001), and forget about appointments (OR: 0.21, 95% CIs [0.09, 0.49]; *p* < 0.001).

**Conclusions:**

This study provides suggestive data on characteristics that might be associated with not attending AAA screening in London. The study design limitations mean that further work is required to evaluate these characteristics more reliably.

## Background

Abdominal aortic aneurysms (AAAs) account for 5000 deaths a year in the UK.^1^ They are most common among men over the age of 65 years, accounting for one in seventy-five deaths within this demographic.^1^

Screening men aged 65, via a one-off abdominal ultrasound, can reduce deaths from AAA by detecting aneurysms before rupture, allowing surveillance and surgical intervention to take place.^2^ It is estimated that AAA screening could prevent half of all AAA-related deaths if uptake were 100%.^3^

In 2021–2022, uptake of AAA screening in the UK was 70.3%.^4^ Uptake was lowest in London, where only 60.2% of invitees attended an appointment.^4^ This disparity is reflective of wider inequalities in screening, with breast, bowel and cervical cancer screening programmes all having lower rates in the capital, compared with the rest of the country.^5^

To date, there has been little research exploring patient barriers and facilitators to AAA screening. The authors are aware of just three studies,^6–8^ only one of which was conducted in England.^6^ That study was conducted in Birmingham,^6^ which is distinct from London in a number of ways, including its ethnic composition and socioeconomic deprivation (both of which have been found to be predictors of uptake in studies exploring demographic variation).^9,10^

The aim of this research, therefore, was to explore the barriers and facilitators to attending AAA screening in London, specifically, with the wider aim of identifying potential strategies to reduce inequalities between the capital and the rest of the UK.

## Method

### Study design and population

To identify barriers to AAA screening in London, we conducted a cross-sectional survey. The study took place between January and March 2023. Data collection continued until August 2023. Eligible patients were men, aged 65 years, who were registered with one of 401 general practices (GPs) in North or South London. Eligible adults were identified using the national IT system for AAA screening (‘SMaRT’). All non-attenders received the survey alongside the ‘Did not attend letter’, which is sent following non-attendance at two consecutive appointments (people who do not attend are offered a second appointment). A randomly selected subset of attenders, meanwhile, were sent a link (via text message) to complete the survey online. The survey was distributed to 2792 non-attenders and 1419 attenders.

### Primary outcome

The primary outcome was attendance at AAA screening, which was assessed via self-report, using the following item: ‘Did you attend an AAA screening appointment?’, with response options ‘Yes’ and ‘No’.

### Measures

Additional items assessed demographic characteristics, general health and possible psychological and practical barriers to AAA screening.

Demographic items included *ethnicity* (categorised as ‘White British/Irish’ and ‘Any other ethnic group’) and *educational attainment* (categorised as ‘GCSE, O-Level, BTEC or below’, and ‘A-level or higher’) (see Appendix 2 in the supplementary material for a comprehensive overview of the survey items and response options).

General health items, meanwhile, included *self-reported health* (dichotomised as ‘Very poor – fair’ and ‘Good – Excellent’), *smoking status* (dichotomised as ‘Never smoked’ and ‘Current or former smoker’), *body mass index* (BMI; derived from self-reported ‘height’ and ‘weight’; dichotomised as ‘BMI < 25’ or ‘BMI > 25’), *co-morbidities* (dichotomised as ‘none’ or ‘one or more’), and *engagement with health services* (derived from date of last NHS appointment; dichotomised as ‘<3 months’ and ‘>3 months’).

Psychological and practical barriers and facilitators were derived from previous literature exploring barriers and facilitators to AAA screening:^6–8^ *fear* (‘It was scary to think what the AAA test might find’), *knowledge* (‘AAA screening is only useful for people with symptoms’; ‘AAA does not usually have any symptoms’’), *perceived benefits* (‘Screening does not lower my chances of dying from AAA’; ‘AAA screening greatly reduces the chances of aneurysms causing serious problems’), *perceived test accuracy* (‘The scan used to find aneurysms is very reliable’) and *practical issues* (‘I find it difficult to make time for medical appointments’; ‘I find it difficult to get to medical appointments’; ‘I have other more important medical problems to worry about’; ‘I cannot afford to travel to/attend medical appointments’, ‘I have caring responsibilities that take priority’; ‘I often forget about appointments’ and ‘I am worried about COVID’).

Agreement with each statement was assessed using a five-point Likert Scale, with the following response options: ‘*Strongly disagree’, ‘Disagree’, ‘Neither agree nor disagree’, ‘Agree’, ‘Strongly agree*’. For the purposes of the analysis, responses were dichotomised as ‘agree’ (‘*Strongly agree*’ and ‘*Agree*’) and ‘disagree’ (‘*Strongly disagree*’, ‘*Disagree*’ and ‘*Neither agree nor disagree*’).

### Analysis

Descriptive statistics were used to report the demographic characteristics of the sample, as well as the means (continuous variables) and frequencies (categorical variables) of survey responses. Associations between demographic characteristics, health items and possible barriers and facilitators, with attendance, were assessed using univariate logistic regression (multivariate logistic regression was not possible, due to low cell counts for multiple items). Associations were considered ‘statistically significant’ if the *p* value was ≤0.05. All analyses were performed using SPSS statistics (Version 27.0).

### Missing data

The number of cases with missing data (including those who responded ‘Prefer not to say’) for each variable was reported using descriptive statistics. The total number of cases included in each analysis is reported in the tables.

## Results

### Sample characteristics and response rate

Of 4211 patients who sent a survey, 270 (6.41%) completed and returned the survey. The majority were White British (*n* = 171, 63.3%), had obtained an A-level or higher qualification (*n* = 163, 60.4%), and considered themselves to be in good-to-excellent health (*n* = 211, 78.1%), despite the majority having a BMI > 25 (*n* = 187, 69.3%), or one or more co-morbidities (*n* = 186, 68.9%), and nearly half being current or former smokers (*n* = 130, 48.1%) (see Appendix Table 1 in the supplementary material).

#### Attendance

Overall, attendance among survey responders was high, with 88.1% (238/270) having attended their appointment. The odds ratios (ORs), 95% confidence intervals (95%CIs) and p values for each variable included in the survey are recorded in Appendix Table 2 in the supplementary material. The ORs, 95% CIs and *p* values for those variables associated with attendance (i.e., *p* < 0.05) are shown in Table 1.

**Table 1.** Factors associated with attendance at AAA screening.

	Attendance, *n* (%)	OR [95% CIs]
*Demographic factors*		
BMI		
<25	60.2 (78.2)	1.00
>25	181.6 (92.7)	2.72 [1.15, 6.46]*
Co-morbidities		
None	67.2 (80.0)	1.00
1 or more	174.6 (93.9)	3.82 [1.63, 8.98]**
Last NHS appointment		
<3 months	166 (91.2)	1.00
>6 months	71 (83.5)	0.41 [0.18, 0.94]*
*Psychological factors (agree/strongly agree)*		
AAA screening is only useful for people with symptoms		
Neither agree nor disagree, disagree, strongly disagree	194 (91.9)	1.00
Agree/strongly agree	47.8 (81.0)	0.37 [0.16, 0.89]*
I find it difficult to make time for medical appointments		
Neither agree nor disagree, disagree, strongly disagree	194 (93.2)	1.00
Agree/strongly agree	47.8 (77.4)	0.25 [0.10, 0.60]**
I find it difficult to get to medical appointments		
Neither agree nor disagree, disagree, strongly disagree	168 (92.3)	1.00
Agree/strongly agree	73.2 (83.8)	0.40 [0.17, 0.91]*
I have other more important medical problems to worry about		
Neither agree nor disagree, disagree, strongly disagree	198.6 (92.6)	1.00
Agree/strongly agree	43.2 (77.7)	0.28 [0.12, 0.64]**
I cannot afford to travel to/attend medical appointments		
Neither agree nor disagree, disagree, strongly disagree	218.6 (92.8)	1.00
Agree/strongly agree	23.2 (67.4)	0.16 [0.07, 0.38]***
I need to get help from friends/family to get to appointments		
Neither agree nor disagree, disagree, strongly disagree	208.6 (91.7)	1.00
Agree/strongly agree	33.2 (78.3)	0.33 [0.13, 0.86]*
I have caring responsibilities that take priority		
Neither agree nor disagree, disagree, strongly disagree	214.8 (93.3)	1.00
Agree/strongly agree	27 (67.5)	0.15 [0.06, 0.34]***
I often forget about appointments		
Neither agree nor disagree, disagree, strongly disagree	205.8 (85.1)	1.00
Agree/strongly agree	36 (73.4)	0.21 [0.09, 0.49]***

Note: OR: odds ratio; 95% CI: 95% confidence interval. *p < .05; **p < .01; ***p < .001.

#### Demographic and health-related predictors of attending AAA screening

The results of the univariate regression analyses (see Appendix Table 2 in the supplementary material) revealed that attendance was significantly higher among those with a BMI > 25 and those with one or more comorbidities; but lower among those who had attended an NHS appointment within the last six months.

#### Psychological and practical barriers to attending AAA screening

Attendance was also significantly lower among those who agreed or strongly agreed with the following statements: ‘AAA screening is only useful for people with symptoms’, ‘I find it difficult to make time for medical appointments’, ‘I find it difficult to get to medical appointments’, ‘I have other more important medical problems to worry about’, ‘I cannot afford to travel to/attend medical appointments’, ‘I need to get help from friends/family to get to appointments’, ‘I have caring responsibilities that take priority’, and ‘I often forget about appointments’.

## Discussion

### Summary and interpretation of main findings

This study explored patient barriers and facilitators to AAA screening in London. With regards to demographic and health-related factors, several characteristics were found to be associated with increased odds for attendance, including increased BMI (i.e., >25), having one or more comorbidities, and having attended an NHS appointment in the past six months. For psychological and practical factors, several characteristics were found to be associated with decreased odds for attendance, including: believing AAA screening is only for people with symptoms, finding it difficult to make time for medical appointments, finding it difficult to get to medical appointments, having more important medical conditions to worry about, not being able to afford to get to/attend medical appointments, needing help from friends/family to get to medical appointments having caring responsibilities that take priority, and forgetting about appointments.

### Comparisons with existing literature

The findings of this study are consistent with those exploring barriers to AAA screening in other settings. For example, a study conducted in Birmingham (England) also found that inconvenient appointment times/locations, and a lack of awareness and understanding of AAA screening, were barriers to attendance.^6^ Likewise, two studies from the Netherlands found that those living further away were less likely to attend, which is consistent with the practical barriers identified in our study.^7,8^ Our findings build on the findings of the previous literature by exploring barriers to AAA screening in London, specifically.

### Interpretation of results

The results of this study highlight a number of populations who are less likely to attend AAA screening, as well as a number of barriers and facilitators to attending AAA screening. Interventions are needed to improve uptake among those who do not regularly attend NHS appointments, and this might include pre-invitation letters explaining the purpose of screening, and online booking systems for rescheduling appointments. Work with patient and public representatives would help determine this further.

The results of this study also suggest that some populations may not perceive themselves as being ‘at risk’, due to their comparatively healthy status. Interventions seeking to improve uptake should consequently emphasise that screening is for apparently healthy people, who may have an asymptomatic health issue.

### Strengths and limitations

This study has several strengths. First, the survey was administered 0–9 weeks after the participant's AAA screening invitation, minimising recall bias. Second, the survey was completed by an ethnically diverse cohort of participants (one-third of responders were of a non-White British ethnicity/nationality), increasing the generalisability of the findings.

This study also has several important limitations. First, the response rate to the questionnaire was very low, and likely to reflect self-selection bias because the uptake of AAA screening in the sample was higher than expected for London. Second, the small number of non-attenders who participated in the survey (potentially, in part, due to inaccurate data on patients’ home address, derived from GP systems) may not be sufficiently representative of non-attenders, and it was not possible to conduct multivariable analyses that can adjust for several factors simultaneously. Finally, attendance was measured via self-report, and thereby subject to self-report bias.

## Conclusion

This study provides suggestive data on characteristics that might be associated with not attending AAA screening in a London population, such as they may not see themselves as being at risk of AAA, due to their perceived health status. However, the study size and likely biases mean that further work is required in a London population to evaluate these characteristics more reliably.

## Supplemental Material

sj-pdf-1-msc-10.1177_09691413241276187 - Supplemental material for Barriers and facilitators of abdominal aortic aneurysm screening in London: A cross-sectional survey Supplemental material, sj-pdf-1-msc-10.1177_09691413241276187 for Barriers and facilitators of abdominal aortic aneurysm screening in London: A cross-sectional survey by Ellie McKay, Joy Wong, Stella Ward, Josephine Ruwende and Robert Kerrison in Journal of Medical Screening

sj-docx-2-msc-10.1177_09691413241276187 - Supplemental material for Barriers and facilitators of abdominal aortic aneurysm screening in London: A cross-sectional survey Supplemental material, sj-docx-2-msc-10.1177_09691413241276187 for Barriers and facilitators of abdominal aortic aneurysm screening in London: A cross-sectional survey by Ellie McKay, Joy Wong, Stella Ward, Josephine Ruwende and Robert Kerrison in Journal of Medical Screening

sj-docx-3-msc-10.1177_09691413241276187 - Supplemental material for Barriers and facilitators of abdominal aortic aneurysm screening in London: A cross-sectional survey Supplemental material, sj-docx-3-msc-10.1177_09691413241276187 for Barriers and facilitators of abdominal aortic aneurysm screening in London: A cross-sectional survey by Ellie McKay, Joy Wong, Stella Ward, Josephine Ruwende and Robert Kerrison in Journal of Medical Screening

sj-docx-4-msc-10.1177_09691413241276187 - Supplemental material for Barriers and facilitators of abdominal aortic aneurysm screening in London: A cross-sectional survey Supplemental material, sj-docx-4-msc-10.1177_09691413241276187 for Barriers and facilitators of abdominal aortic aneurysm screening in London: A cross-sectional survey by Ellie McKay, Joy Wong, Stella Ward, Josephine Ruwende and Robert Kerrison in Journal of Medical Screening
