# Supplementary material for: Barriers and facilitators of abdominal aortic aneurysm screening in London: A cross-sectional survey
Source: J Med Screen. 2024 Aug 23;32(1):53–6. doi: 10.1177/09691413241276187 (PMC11869502; doi:10.1177/09691413241276187)
Supplement: sj-docx-2-msc-10.1177_09691413241276187 - Supplemental material for Barriers and facilitators of abdominal aortic aneurysm screening in London: A cross-sectional survey [file sj-docx-2-msc-10.1177_09691413241276187.docx]

<Title <First Name> <Last name>

<Address Line 1>
<Address Line 2>
<Address Line 3>
<Address Line 4>

<DD Month Year>

Dear <Title> <First Name> <Last Name>

**Survey relating to Abdominal Aortic Aneurism (AAA) Screening**

To help us to deliver our vision of ‘Five Star’ patient care to our communities, we are supporting several national projects which aim to improve the care of our patients.

You have recently been invited by your local screening programme to attend Abdominal Aortic Aneurism (AAA) Screening.

The survey enclosed has been designed to help us understand more about your experience of being invited for this test. It would be very helpful for future patients if you could share your views by completing this survey. You can complete either the paper questionnaire or use the QR code to complete online.

We are aware that not everyone who is asked goes on to attend AAA screening. If you did not, you may still be able to help us. Please complete the survey, to help us understand how the programme can be improved.

Thank you for your support with this important project.

Yours sincerely

Dr Josephine Ruwende
Consultant in Public Health
NHS England


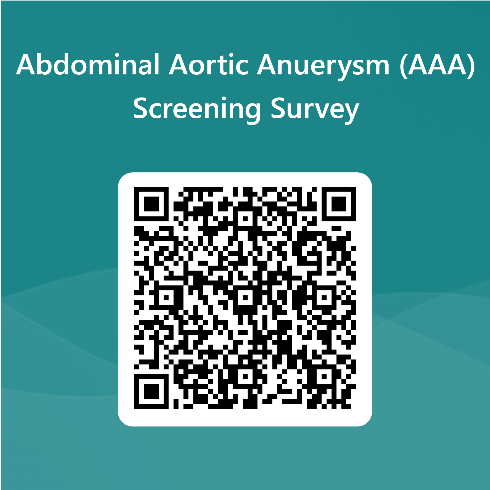


**ABDOMINAL AORTIC ANEURYSM (AAA) SCREENING SURVEY**

**ABOUT YOU**

| **Q1.** Which of these best describes your ethnic group? | | | | | | | | | | |
| --- | --- | --- | --- | --- | --- | --- | --- | --- | --- | --- |
|  | **White** |  | **Mixed** |  | **Asian or Asian British** |  | **Black or Black British** |  | **Chinese/ Other** |  |
| □ | White British | □ | White and Black Caribbean | □ | Indian | □ | Black Caribbean | □ | Chinese |  |
| □ | White Irish | □ | White and Black African | □ | Pakistani | □ | Black African | □ | Other… |  |
| □ | Any other White background | □ | White and Asian | □ | Bangladeshi | □ | Any other Black background | □ | Prefer not to say |  |
|  |  | □ | Any other mixed background | □ | Any other Asian background |  |  |  |  |  |

| **Q2.** What is the highest level of education qualification you have obtained? | | | |
| --- | --- | --- | --- |
| □ | Degree or higher degree | □ | O level or GCSE (Grade A - C) |
| □ | Higher education qualification below degree level | □ | O level or GCSE (Grade D – G) |
| □ | A-levels or higher | □ | No formal qualification |
| □ | ONC/BTEC | □ | Other (please specify):__________________ |
| □ | Still studying | □ | Prefer not to say |

| **Q3.** What are your height and weight? | | |
| --- | --- | --- |
| Height: _____ ft. _____ inches **OR** ________ cm  Weight: _____ stone _____ pounds **OR** ________kgs | □ | Prefer not to say |

| **Q4.** Do you smoke or use tobacco products? | | | | | |
| --- | --- | --- | --- | --- | --- |
| □ | Never smoked | □ | Ex-smoker | □ | Smoker |

| **Q5.** What are the first three digits of your postcode:  ….................. | □ | Prefer not to say |
| --- | --- | --- |

**YOUR HEALTH**

| **Q6.** Overall, how would you rate your health? | | | | | | | | | | | |
| --- | --- | --- | --- | --- | --- | --- | --- | --- | --- | --- | --- |
| □ | Excellent | □ | Very good | □ | Good | □ | Fair | □ | Poor | □ | Very poor |

| **Q7.** When did you last visit an NHS or healthcare service (e.g. dentist, GP, hospital, vaccine etc.) | | | |
| --- | --- | --- | --- |
| □ | Last 3 months | □ | Over a year ago |
| □ | Last 6 months | □ | Prefer not to say/unsure |

| **Q8.** Have you ever been told by a doctor that you have any of the following conditions?  (Please tick all that apply) | | | |
| --- | --- | --- | --- |
| □ | COPD (‘Chronic Obstructive Pulmonary Disease’) | □ | High blood pressure |
| □ | Diabetes | □ | High cholesterol |
| □ | Connective tissue disorder (e.g. Marfan Syndrome, Ehlers-Danlos Syndrome, etc.) | □ | Heart disease/ CVD (‘Cardiovascular Disease’) |

| **Q9.** Have any of the following people ever been diagnosed with an **Abdominal Aortic Aneurysm**?  (Please tick all that apply) | | | |
| --- | --- | --- | --- |
| □ | Wife / Husband / Partner | □ | Family member (blood relative) |
| □ | Family member (non-blood relative) | □ | Close friend |

**YOUR AAA SCREENING INVITE**

| **Q10.** Do you remember receiving an invitation for AAA screening? | | | |
| --- | --- | --- | --- |
| □ | Yes (go to **Q11**) | □ | No / don’t remember (go to **Q14**) |

| **Q11.** When you received the invitation, how interested were you in having the AAA screening test? | | | | | | | |
| --- | --- | --- | --- | --- | --- | --- | --- |
| □ | Very | □ | Moderately | □ | Somewhat | □ | Not at all |

| **Q12.** After reading your invitation, what did you do? | | | | | |
| --- | --- | --- | --- | --- | --- |
| □ | I changed my appointment date or time | □ | I cancelled my appointment | □ | Nothing |

| **Q13.** Did you attend a AAA screening appointment? | | | |
| --- | --- | --- | --- |
| □ | Yes | □ | No |

| **Q14.** Please tell us how much you agree or disagree with the following statements: | | | | | |
| --- | --- | --- | --- | --- | --- |
|  | Strongly agree | Somewhat agree | Neither agree nor disagree | Somewhat disagree | Strongly disagree |
| It was scary to think what the AAA test might find | □ | □ | □ | □ | □ |
| AAA screening is only useful for people with symptoms | □ | □ | □ | □ | □ |
| Screening does not lower my chances of dying from AAA | □ | □ | □ | □ | □ |
| AAA does not usually have any symptoms | □ | □ | □ | □ | □ |
| AAA screening greatly reduces the chances of aneurysms causing serious problems. | □ | □ | □ | □ | □ |
| The scan used to find aneurysms is very reliable | □ | □ | □ | □ | □ |

| **Q15.** Please tell us how much you agree or disagree with the following statements: | | | | | |
| --- | --- | --- | --- | --- | --- |
|  | Strongly agree | Somewhat agree | Neither agree nor disagree | Somewhat disagree | Strongly disagree |
| I find it difficult to make time for medical appointments | □ | □ | □ | □ | □ |
| I find it difficult to get to medical appointments | □ | □ | □ | □ | □ |
| I have other more important medical problems to worry about | □ | □ | □ | □ | □ |
| I cannot afford to travel to / attend medical appointments | □ | □ | □ | □ | □ |
| I need help from friends / family to get to appointments | □ | □ | □ | □ | □ |
| I have caring responsibilities that take priority | □ | □ | □ | □ | □ |
| I often forget about appts. | □ | □ | □ | □ | □ |
| I am worried about COVID | □ | □ | □ | □ | □ |

**Q16.**
Please include any other reasons for not attending AAA screening:

**Q17.** Is there anything else that would make you more likely to attend AAA screening?

**FURTHER INFORMATION**

We are looking to recruit a small number of patients for an interview about the AAA screening programme. Would you be willing to take part in an online/telephone interview (you do not need to have attended AAA screening to take part in an interview)?

**Yes □ No □**

If you ticked ‘YES’, please provide your details below (this information will be treated in the strictest confidence):

Name__________________________________________

Address________________________________________

_______________________________________________

_______________________________________________

Email___________________________________________

Telephone_______________________________________

**THANK YOU**

Thank you very much for taking the time to complete the questionnaire. Your answers are very important to us.

Please return this questionnaire in the enclosed freepost envelope.

No stamp is required.

| If you lose the freepost envelope, please post to: FREEPOST  Wellington House 133-155 Waterloo Rd London SE1 8UG (AAA SCREENING SURVEY) |
| --- |

The return of a completed questionnaire is confirmation of your consent to take part in the survey and allows the team to use the personal information you provide in their activities.

All data you provide to the team will be treated in the strictest confidence and will be stored securely in accordance with the Data Protection Act 1998 and the General Data Protection Regulation 2017.
