# Supplementary material for: Barriers and facilitators of abdominal aortic aneurysm screening in London: A cross-sectional survey
Source: J Med Screen. 2024 Aug 23;32(1):53–6. doi: 10.1177/09691413241276187 (PMC11869502; doi:10.1177/09691413241276187)
Supplement: sj-docx-3-msc-10.1177_09691413241276187 - Supplemental material for Barriers and facilitators of abdominal aortic aneurysm screening in London: A cross-sectional survey [file sj-docx-3-msc-10.1177_09691413241276187.docx]

| **Appendix Table 1.** Sample characteristics. | |
| --- | --- |
|  | n (%) |
| Total | 270 |
| Demographic characteristics | |
| Ethnicity | |
| White British / Irish | 171 (63.3) |
| Any other ethnic group | 90 (33.3) |
| Missing | 9 (3.3) |
| Education | |
| GCSE, O-Level, BTEC or below | 69 (25.6) |
| A-level or higher | 163 (60.4) |
| Missing | 38 (14.1) |
| Self-reported health | |
| Very poor – fair | 52 (19.3) |
| Good - Excellent | 211 (78.1) |
| Missing | 7 (2.6) |
| Smoking status | |
| Never smoked | 136 (50.4) |
| Current or former smoker | 130 (48.1) |
| Missing | 4 (1.5) |
| BMI | |
| <25 | 69 (25.6) |
| >25 | 187 (69.3) |
| Missing | 14 (5.2) |
| Co-morbidities | |
| None | 84 (31.1) |
| 1 or more | 186 (68.9) |
| Missing | 0 |
| Last NHS appointment | |
| <3 months | 182 (67.4) |
| >3 months | 85 (31.5) |
| Missing | 3 (1.1) |
