# Supplementary material for: Barriers and facilitators of abdominal aortic aneurysm screening in London: A cross-sectional survey
Source: J Med Screen. 2024 Aug 23;32(1):53–6. doi: 10.1177/09691413241276187 (PMC11869502; doi:10.1177/09691413241276187)
Supplement: sj-docx-4-msc-10.1177_09691413241276187 - Supplemental material for Barriers and facilitators of abdominal aortic aneurysm screening in London: A cross-sectional survey [file sj-docx-4-msc-10.1177_09691413241276187.docx]

| **Appendix Table 2.** Factors potentially associated with attendance at AAA screening | | |
| --- | --- | --- |
|  | Attendance, n (%) | OR (95% CIs) |
| Demographic factors | | |
| Ethnicity | | |
| White British / Irish | 160.6 (92.6) | 1.00 |
| Any other ethnic group | 81.2 (84.1) | 0.42 (0.17, 1.04) |
| Education | | |
| GCSE, O-Level, BTEC or below | 79.4 (87.8) | 1.00 |
| A-level or higher | 162.4 (90.4) | 1.31 (0.53, 3.25) |
| Self-reported health | | |
| Very poor – fair | 46.4 (85.3) | 1.00 |
| Good - Excellent | 195.4 (90.6) | 1.66 (0.60, 4.63) |
| Smoking status | | |
| Never smoked | 120.4 (87.1) | 1.00 |
| Current or former smoker | 121.4 (92.1) | 1.73 (0.74, 4.05) |
| BMI | | |
| <25 | 60.2 (78.2) | 1.00 |
| >25 | 181.6 (92.7) | **2.72 (1.15, 6.46)*** |
| Co-morbidities | | |
| None | 67.2 (80.0) | 1.00 |
| 1 or more | 174.6 (93.9) | **3.82 (1.63, 8.98)**** |
| Last NHS appointment | | |
| <3 months | 166 (91.2) | 1.00 |
| >6 months | 71 (83.5) | **0.41 (0.18, 0.94)*** |
| Psychological factors (Agree / Strongly agree) | | |
| It was scary to think what the AAA test might find | | |
| Neither agree nor disagree, disagree, strongly disagree | 142.6 (92.5) | 1.00 |
| Agree / Strongly agree | 99.2 (85.7) | 0.48 (0.20, 1.15) |
| AAA screening is only useful for people with symptoms | | |
| Neither agree nor disagree, disagree, strongly disagree | 194 (91.9) | 1.00 |
| Agree / Strongly agree | 47.8 (81.0) | **0.37 (0.16, 0.89)*** |
| Screening does not lower my chances of dying from AAA | | |
| Neither agree nor disagree, disagree, strongly disagree | 163.8 (91.8) | 1.00 |
| Agree / Strongly agree | 78 (85.2) | 0.51 (0.23, 1.14) |
| AAA does not usually have any symptoms | | |
| Neither agree nor disagree, disagree, strongly disagree | 126.2 (87.5) | 1.00 |
| Agree / Strongly agree | 115.6 (91.9) | 1.63 (0.65, 4.08) |
| AAA screening greatly reduces the chances of aneurysms causing serious problems | | |
| Neither agree nor disagree, disagree, strongly disagree | 61.8 (90.4) | 1.00 |
| Agree / Strongly agree | 180 (89.3) | 0.89 (0.34, 2.33) |
| The scan used to find aneurysms is very reliable | | |
| Neither agree nor disagree, disagree, strongly disagree | 76.7 (87.1) | 1.00 |
| Agree / Strongly agree | 165.2 (90.8) | 1.47 (0.63, 3.39) |
| I find it difficult to make time for medical appointments | | |
| Neither agree nor disagree, disagree, strongly disagree | 194 (93.2) | 1.00 |
| Agree / Strongly agree | 47.8 (77.4) | **0.25 (0.10, 0.60)**** |
| I find it difficult to get to medical appointments | | |
| Neither agree nor disagree, disagree, strongly disagree | 168 (92.3) | 1.00 |
| Agree / Strongly agree | 73.2 (83.8) | **0.40 (0.17, 0.91)*** |
| I have other more important medical problems to worry about | | |
| Neither agree nor disagree, disagree, strongly disagree | 198.6 (92.6) | 1.00 |
| Agree / Strongly agree | 43.2 (77.7) | **0.28 (0.12, 0.64)**** |
| I cannot afford to travel to / attend medical appointments | | |
| Neither agree nor disagree, disagree, strongly disagree | 218.6 (92.8) | 1.00 |
| Agree / Strongly agree | 23.2 (67.4) | **0.16 (0.07, 0.38)***** |
| I need to get help from friends / family to get to appointments | | |
| Neither agree nor disagree, disagree, strongly disagree | 208.6 (91.7) | 1.00 |
| Agree / Strongly agree | 33.2 (78.3) | **0.33 (0.13, 0.86)*** |
| I have caring responsibilities that take priority | | |
| Neither agree nor disagree, disagree, strongly disagree | 214.8 (93.3) | 1.00 |
| Agree / Strongly agree | 27 (67.5) | **0.15 (0.06, 0.34)***** |
| I often forget about appointments | | |
| Neither agree nor disagree, disagree, strongly disagree | 205.8 (85.1) | 1.00 |
| Agree / Strongly agree | 36 (73.4) | **0.21 (0.09, 0.49)***** |
| I am worried about COVID | | |
| Neither agree nor disagree, disagree, strongly disagree | 204 (91.0) | 1.00 |
| Agree / Strongly agree | 37.8 (82.5) | 0.47 (0.18, 1.22) |
